# Long-Term Hydraulic Adjustment of Three Tropical Moist Forest Tree Species to Changing Climate

**DOI:** 10.3389/fpls.2018.01761

**Published:** 2018-12-04

**Authors:** Mahmuda Islam, Mizanur Rahman, Achim Bräuning

**Affiliations:** ^1^Department of Geography and Geosciences, Institute of Geography, Friedrich-Alexander University Erlangen-Nuremberg, Erlangen, Germany; ^2^Department of Forestry and Environmental Science, Shahjalal University of Science and Technology, Sylhet, Bangladesh

**Keywords:** Bangladesh, hydraulic conductivity, tropical forests, climate change, hydraulic safety, xylem anatomy, safety-efficiency trade-off

## Abstract

Xylem hydraulic adjustment to global climatic changes was reported from temperate, boreal, and Mediterranean tree species. Yet, the long-term hydraulic adjustment in tropical tree species has not been studied so far. Here we developed the first standard chronologies of three hydraulic trait variables for three South Asian moist forest tree species to analyze their long-term hydraulic responses to changing climate. Based on wood anatomical measurements, we calculated Hagen–Poiseuille hydraulically weighted vessel diameter (D_H_), potential specific hydraulic conductivity (K_S_), and vulnerability index (V_X_) and developed standard chronologies of these variables for *Chukrasia tabularis, Toona ciliata*, and *Lagerstroemia speciosa* which are different in their xylem structure, wood density, shade tolerance, growth rates, and habitat preferences. Bootstrap correlation analysis revealed that vapor pressure deficit (VPD) strongly positively influenced the xylem water transport capacity in *C. tabularis*, whereas *T. ciliata* was affected by both temperature and precipitation. The hydraulic conductivity of *L. speciosa* was mainly affected by temperature. Different adjustment strategies were observed among the species, probably due to the differences in life history strategies and xylem properties. No positive relationship of conductivity and radial growth was found, but a trade-off between hydraulic safety and efficiency was observed in all studied species.

## Introduction

The xylem in woody plants serves multiple function such as water and nutrient transport, mechanical support, maintaining stomatal opening and carbon uptake (Carlquist, 2001; Sperry et al., 2003). However, safe and efficient water transport from root to leaf is considered as one of the vital functions (Brodribb and Hill, 1999; Sperry et al., 2003) which determines plant performance, especially during stressful environmental conditions (Tyree and Sperry, 1989; Larcher, 2003; Fonti et al., 2010). Global environmental changes such as rising temperature, changes in temporal precipitation patterns, frequent, and extreme drought events (IPCC, 2014) may have substantial impact on plant growth, phenology, physiology, and other developmental processes (Ceulemans et al., 1997; Crous et al., 2011; Nitschke et al., 2017; He et al., 2018). In order to cope with changing environment particularly related to water availability, vascular plants may adjust their hydraulic architecture so that they can maximize water transportation and can minimize the risk of hydraulic failure (Sperry et al., 2002; Fonti et al., 2010; Martinez-Vilalta et al., 2014). For example, plants produce a small number of large size vessels in response to drought (Hacke et al., 2006; Sperry et al., 2006; Wheeler, 2011; Haworth et al., 2017). This phenotypic plasticity allows plants efficient transportation of water from the root to the leaf (Zimmermann, 1983) since the water flow along a vessel is proportional to the fourth power of its radius according to the Hagen-Poiseuille Law (Tyree and Zimmermann, 2002; Sperry et al., 2006; Steppe and Lemeur, 2007; McCulloh et al., 2010). Likewise, as a resopnse to avoid hydraulic failure due to cavitation, vessel frequency is increased so that a higher number of smaller vessels remain functional in terms of cavitation (Sperry et al., 2008; Zanne et al., 2010; Venturas et al., 2017; Pérez-de-Lis et al., 2018).

The hydraulic adjustment mechanism has been investigated across tree populations, species and at different spatial scales (Borghetti et al., 2017). However, less attention has been paid to get insight into the long-term temporal plasticity particularly in tropical forest trees although tropical forests are supposed to be more vulnerable to climate change as expected before (Clark et al., 2003; Doughty and Goulden, 2009). Studying temporal plasticity in xylem hydraulic structure in response to environmental changes requires developing chronologies of hydraulic traits from long-term wood anatomical time series. Particularly, the necessity to develop time series of hydraulic traits has been emphasized to study climate change adaptability both in temperate (Bryukhanova and Fonti, 2013; Oladi et al., 2014; Rita et al., 2015; Schuldt et al., 2016; Noyer et al., 2017; Granda et al., 2018; Pérez-de-Lis et al., 2018) and Mediterranean forests (Corcuera et al., 2004a, 2006; Gea-Izquierdo et al., 2012; Rita et al., 2016; Castagneri et al., 2017; Martínez-Sancho et al., 2017). So far, no hydraulic conductivity (K_S_) time series were developed for tropical forest trees. A recent global scale meta-analysis (Borghetti et al., 2017) on the temporal plasticity in xylem hydraulic traits revealed that almost all existing studies were done in high latitude regions (36–52° N), with no studies in tropical and sub-tropical regions (−30° S to 30° N). This points to a clear research gap concerning hydraulic adjustment of tree species in tropical regions.

Tropical broadleaf species are understudied in terms of long-term wood anatomical climate adaptations. *Tectona grandis* was frequently used to develop vessel chronologies (Pumijumnong and Park, 1999; Bhattacharyya et al., 2007; Venegas-González et al., 2015; Sinha et al., 2017). Besides, some mangrove species such as *Heretiera fomes* and *Rhizophora mucronata* were studied to develop wood anatomical time series (Verheyden et al., 2005; Chowdhury et al., 2008). In a preliminary study, we investigated wood anatomical features in 27 tree species from a moist tropical forest in Bangladesh and showed their dendrochronological potential (Islam et al., 2018a). In a further step, we established vessel chronologies of diffuse-porous and intermediate shade tolerant *Chukrasia tabularis* from a Bangladeshi moist tropical forest and showed that the vessel features are highly sensitive to climate (Islam et al., 2018b). Some other studies have shown the potential of developing wood anatomical time series in moist tropical forests (Ohashi et al., 2011, 2014). Nevertheless, time series of hydraulic conductivity of any of the tropical tree species have not been measured so far. Variations of hydraulic conductivity along a water flow path from root to shoot were studied in an Indonesian tropical forest (Kotowska et al., 2015). Also, radial variations of tree hydraulic properties were studied in a Southeast Asian tropical forest (Rungwattana and Hietz, 2017). However, annually resolved time series of hydraulic traits in tropical tree species that can be related to environmental variable are yet to be developed. Moreover, moist tropical forests are rich in species and functional diversity (Slik et al., 2015). It is reported that different functional groups may show different hydraulic adjustment strategy in response to changing environment (Poorter et al., 2010). Several studies suggested that shade intolerant species have higher hydraulic efficiency than shade tolerant species (Markesteijn et al., 2011a,b; Hoeber et al., 2014; Hietz et al., 2017; Rungwattana and Hietz, 2017). Similarly, ring-porous species are likely to be more vulnarable to cavitation than diffuse porous species (Sperry et al., 1994; Taneda and Sperry, 2008; Ogasa et al., 2014).

Interspecific differences in xylem hydraulic architecture (vessel features) may reflect differences in the way of hydraulic adjustment in response to environmental variability (Fonti et al., 2010), providing valuable information about the plasticity a particular species shows under changing environmental conditions. It is therefore important to test the range of hydrosystem adjustment in species of different functional types. In the present study, we developed long-term chronologies of three hydraulic trait variables for three tropical moist forest trees which differ in their xylem anatomy (diffuse porous, semi ring-porous and ring-porous), shade tolerance (shade tolerant, shade intolerant, and partial shade tolerant), wood density, growth rates, and habitat preferences. We aimed at testing the following hypotheses:

▪ Trees modulate their hydraulic traits in response to inter annual variation in climate variables in moist tropical forests.
▪ Phenotypic plasticity differs among functionally different moist forest trees.
▪ Hydraulic conductivity is positively associated with radial growth since it is assumed that efficient water transport may favor tree growth (Poorter et al., 2010; Fan et al., 2012; Schuldt et al., 2016).
▪ Trees of different functional types maintain the well-known trade-off between hydraulic efficiency and safety through their life time under tropical moist environment.

## Materials and Methods

### Study Area and Species

**Figure 1:**
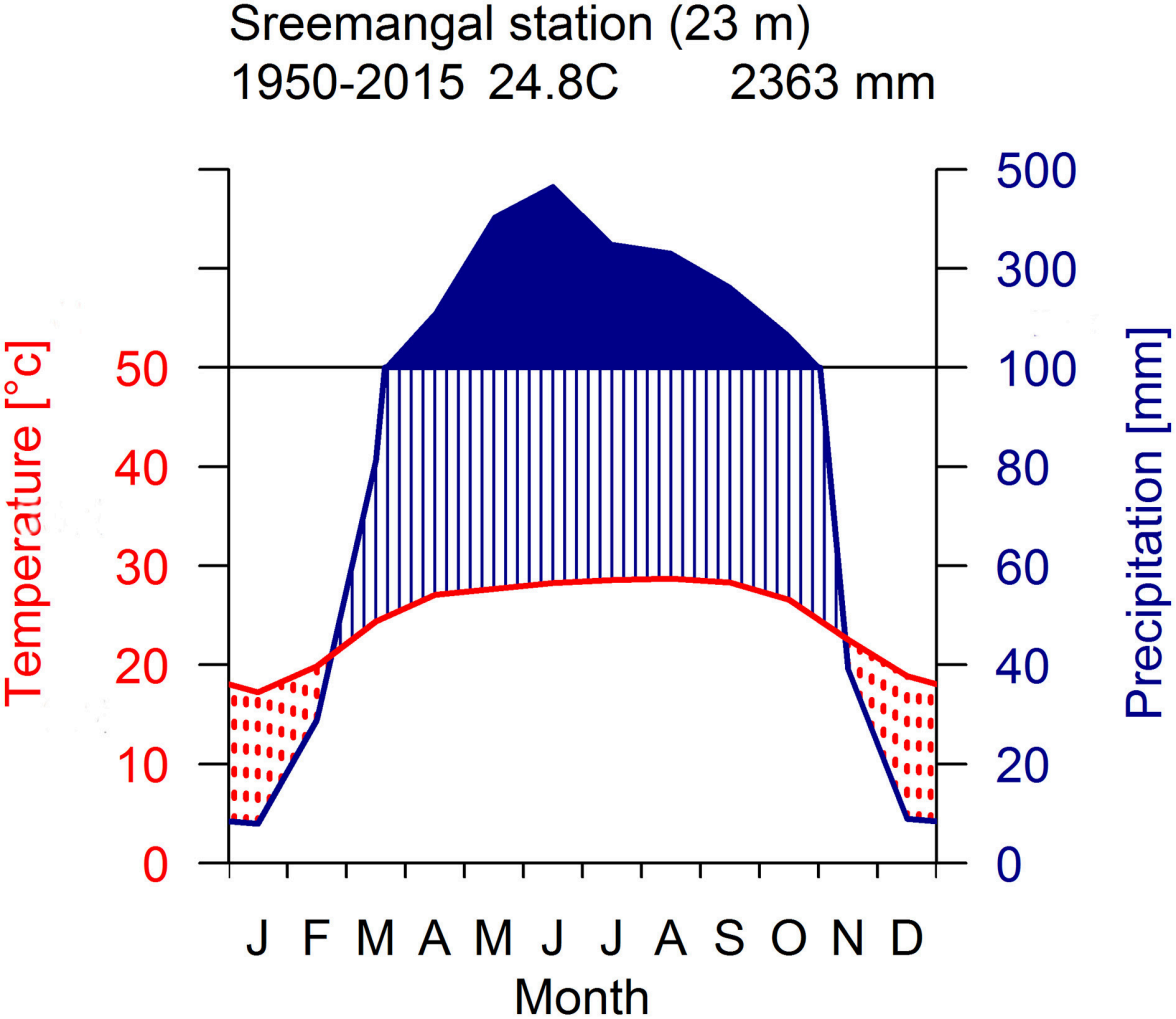
Walter-Leith monthly climate diagram for the study area derived from data collected at meteorological station (Sreemangal, 23 km) for the period 1950–2015.

**Figure 2:**
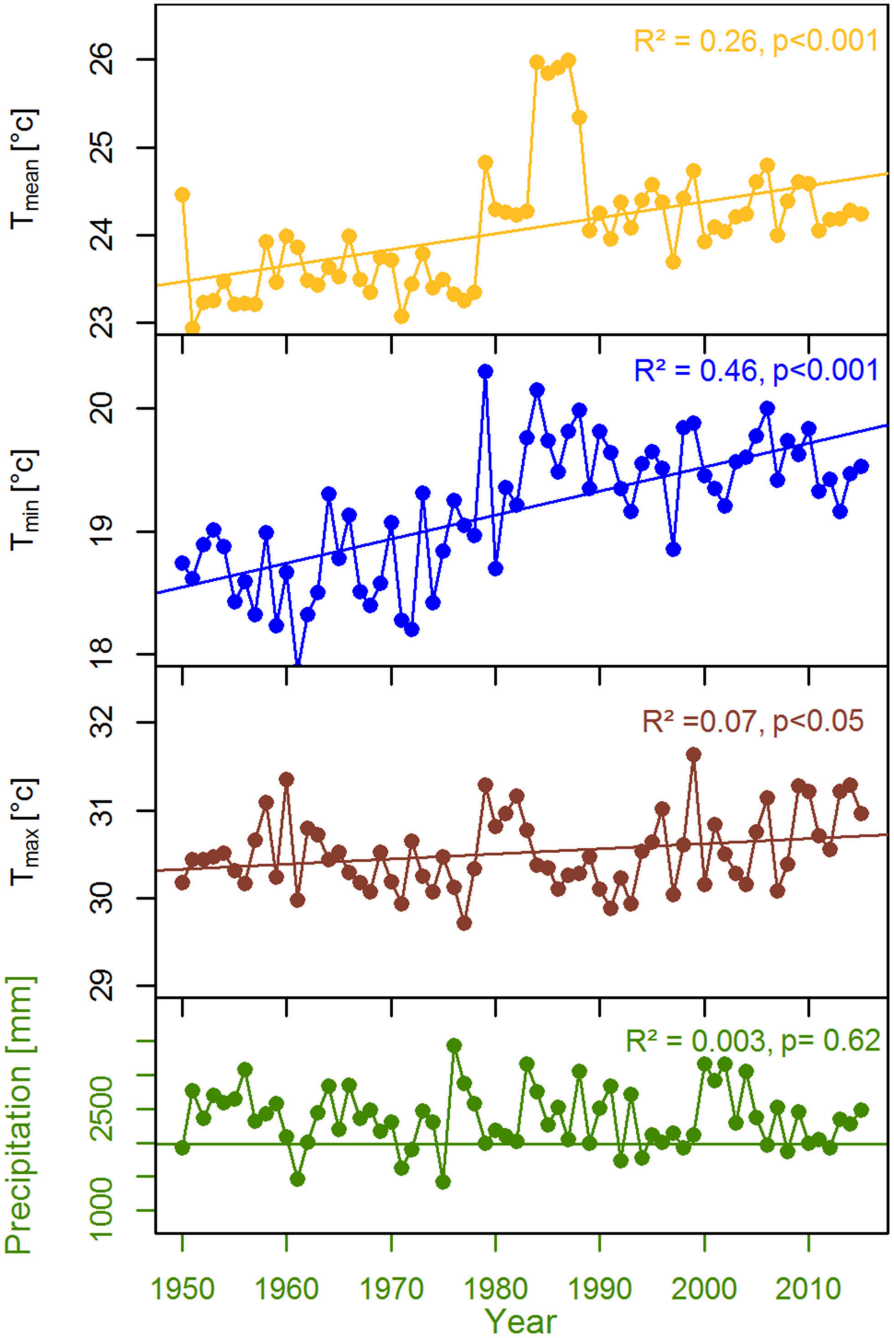
Trends in local climate variables for the period over 1950–2015.

The study was carried out within the Rema-Kalenga forest in the north eastern part of Bangladesh (24°06′−24°14′N, and 91°36′−91°39′E). An area of 1,795 ha in Rema-Kalenga forest was declared as Wildlife Sanctuary which is bounded on the east and south by Tripura state of India. The study sites consist of hilly areas of around 100 m elevation and low-lying valleys. Soils of the forest vary from clay loam on the relatively level ground or valleys to sandy loam on the hills (Hassan, 1994). The climate of the study area is characterized as tropical humid monsoonal climate (Holdridge, 1967). According to the climate data recorded at the nearest climate station (Sreemangal, Bangladesh), the mean annual temperature and total annual precipitation over the period 1950–2015 are 24.8°C and 2,363 mm, respectively (Figure 1). A distinct seasonality is present, with a dry season spanning from November to February (monthly rainfall < 100 mm) (Figure 1). May-August is the main monsoon season, March–April is the pre-monsoon and September-October is the late-monsoon season in our study region. A significant increasing trend was observed in mean, minimum, and maximum temperatures, whereas precipitation did not show any clear trend over 1950–2015 (Figure 2).

The studied forests are recognized for the ecological services they provide and are dominated by deciduous and evergreen trees including *Chukrasia tabularis, Toona ciliata, Syzygium grandis, Terminalia bellirica, Lagerstroemia speciosa, Dillenia pentagyna, Dipterocarpus turbinatus, Tectona grandis*, as well as various species of *Ficus* and *Albizia*. We selected three species (*Chukrasia tabularis, Toona ciliata*, and *Lagerstroemia speciosa*) based on their wide distribution across the tropics, their functional differences, and the characteristics of growth-ring boundaries (Islam et al., 2018a). *C. tabularis* comprises diffuse porous wood anatomy with distinct growth-ring boundaries delineated by marginal parenchyma bands. Xylem anatomy of *T. ciliata* is characterized by semi ring-porous vessel distribution, with distinct growth-ring boundaries defined by marginal parenchyma and large earlywood vessels. *L. speciosa* is characterized by ring-porous wood anatomy with distinct growth-ring boundaries detected by large early wood vessels following a marginal parenchyma band. All three study species were found to form annual growth rings (Bhattacharyya and Yadav, 1989; Heinrich et al., 2009; Vlam et al., 2014; Rahman et al., 2018). A detailed description of the selected species is given in Table 1.

**Table 1:** Characteristics of studied species.

	***Chukrasia tabularis***	***Toona ciliata***	***Lagerstroemia speciosa***
Family	Meliaceae	Meliaceae	Lythraceae
Distribution^a^	SA, EA, SEA	SA, SEA, Af, A	SA, EA, SEA
Phenology^b^^*^	Brevi-deciduous	Deciduous	Deciduous
Ecological guild^c^^*^	PST	P	ST
Max. height (m)	40	40	26
Position in the canopy	Middle	Top	Middle
Xylem structure	Diffuse porous	Semi ring-porous	Ring-porous
Tree-ring boundary	Fairly distinct	Fairly distinct	Fairly distinct
Main anatomical features delineating tree-ring boundary^d^	MP	P, MP, FWT	P, MP, FWT
Main use of wood^e^	F, C	F, V	F, B

a *SA, South Asia; EA, East Asia; SEA, South-East Asia; A, Australia; Af, Africa*.

b *Deciduous: tree leafless for more than 4 weeks*.

c *P, Pioneer; ST, Shade tolerant; PST, Partial shade tolerant*.

d *MP, Marginal parenchyma band (terminal or initial); P, Porosity; FWT, Thick-walled latewood fibers*.

e *F = Furniture; C, Construction; V, Veneer and plywood; B, Boat making*.

* *(Williams et al., 2008; Orwa et al., 2009)*.

### Wood Sample Collection, Preparation, and Anatomical Measurements

We extracted increment cores from 105 trees at breast height (1.3 m) by using a 5.0 mm diameter increment borer (Vantaa, Finland). In the field, plastic holders were used to store the cores immediately after extraction. Cores were air dried for 24 h to avoid any attack by fungi. In the laboratory, increment cores were mounted on wooden holders and sanded with increasingly finer grain paper up to 2,000 grit to make wood anatomical features clearly visible (Stokes and Smiley, 1968). A high-pressure water blast was used to remove wood dust and tyloses inside the vessels. White chalk was applied on the surface to fill the vessels, thus improving the contrast from the bulk tissues. Ring-width (RW) was measured directly on the core surface by using a Lintab 6 measuring system (Rinntech, Heidelberg, Germany) with a precision of 0.01 mm. Ring-width series were visually crossdated using TSAP-Win (Rinntech, Heidelberg, Germany) and statistically evaluated by *t*-test and Gleichläufigkeit values (GLK, sign test; Eckstein and Bauch, 1969). Crossdating quality was finally checked using COFECHA (Grissino-Mayer, 2001). A total of 70 trees were successfully crossdated.

**Figure 3:**
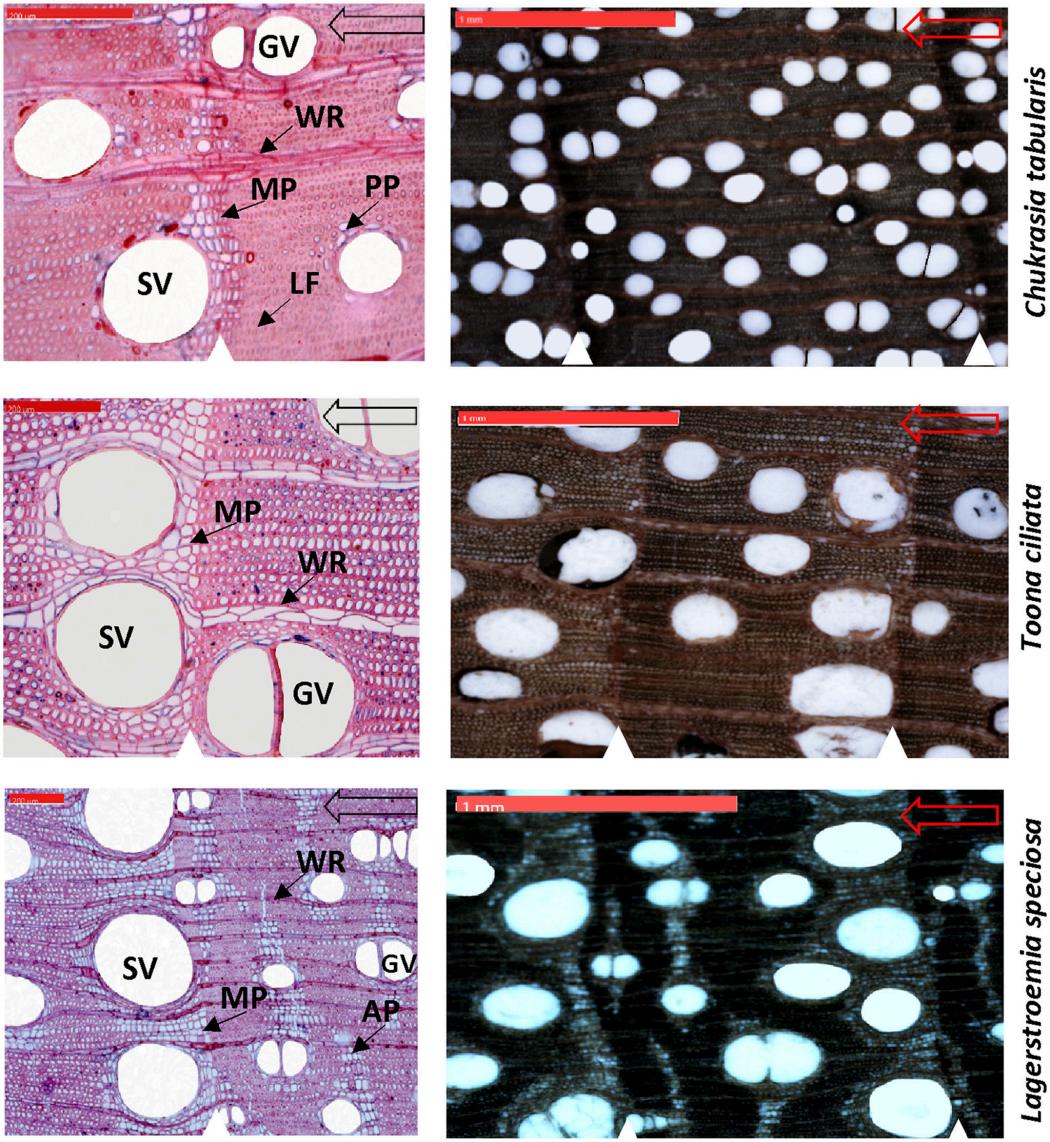
High resolution digital microscopic images of the thin sections (**Left**) and cross sectional wood surface (**Right**) showing characteristic wood anatomical features of three South Asian tropical moist forest tree species. White triangles indicate growth-ring boundaries. Hollow arrows indicate growth direction. SV, solitary vessels; GV, grouped vessels; WR, wood rays; MP, marginal parenchyma; LF, latewood fibers; AP, Apotracheal parenchyma; PP, Paratracheal parenchyma. Scale bars are 200 μm in the thin sections and 1 mm in the cross sectional wood surface images.

Wood anatomical preparation and measurements are laborious and time consuming. Hence, a subset of 9 cross-dated trees from each species (total 27 trees) was finally selected for wood anatomical measurements. We took digital images of the cross sectional surfaces of the cores (5,184 × 3,456 pixels) by using a digital microscope Zeiss Smartzoom 5 (Carl Zeiss Microscopy GmbH 2014, Jena, Germany) for xylem anatomical measurement (Figure 3).To simplify the measurements, Adobe Photoshop was used to further increase the contrast between the vessels and other wood elements and manually edit unclear cases of vessel lumen area due to preparation artifacts. Before the measurement, the image was calibrated from a scale bar of known length in the image. On each image, tree-ring boundaries were identified with their year of formation and an analysis region was created in each tree ring by closing the regions delineated by the ring boundary paths in WinCELL. All vessels (vessel area: 0.002–20.0 mm^2^, other features smaller or larger were discarded) were measured for each dated tree ring using an image–analysis software WinCELL 2012a (Regent Instruments Inc., Québec, Canada) which is specifically designed for wood cell analysis. We measured four vessel parameters including the number of vessels (NV), mean vessel tangential diameter (MVTD), mean vessel radial diameter (MVRD), and mean vessel area (MVA). NV, MVTD, and MVRD were then used to calculate vessel density (VD) and hydraulic traits such as the Hagen–Poiseuille hydraulically weighted vessel diameter (D_H_), potential specific hydraulic conductivity (K_S_), and vulnerability index (V_X_).

### Xylem Hydraulic Traits Calculations

Since most of the vessels in the studied tree species are not circular but mostly elliptical in shape, radial and tangential diameters (Oladi et al., 2014) of each vessel were considered as major (a_i_) and minor (bi) axis. The Hagen–Poiseuille hydraulically weighted D_H_ was computed by applying the following equations (Lewis and Boose, 1995; Tyree and Zimmermann, 2002; Steppe and Lemeur, 2007)

$$\begin{array}{l} D_{H}=\sqrt[{4}]{\frac{1}{n}\overset{n}{\underset{i=1}{\sum }}\frac{{2a}^{2}b^{2}}{a_{i}^{2}+b_{i}^{2}}\;} \end{array}$$

Where n is the number of measured vessels, a and b (μm) are the two axes of the vessels

The potential specific hydraulic conductivity is the total estimated conductivity of a group of vessels assuming that their flow is governed by the Hagen–Poiseuille equation. Potential specific hydraulic conductivity K_S_ (kg m^−1^ s^−1^ Mpa^−1^) was calculated using the Hagen–Poiseuille's law (Tyree and Zimmermann, 2002) as follows:

$$\begin{array}{l} K_{S}=\left(\frac{\pi \;\rho }{128\;\eta }\right)*{D_{H}}^{4}*VD \end{array}$$

Where η is the viscosity of water (1.002 × 10^−9^ MPa s^−1^), ρ the density of water at 20°C (998.21 kg m^3^), VD (mm^−2^) is the ratio of NV per analyzed area (mm^2^), and D_H_ is the Hagen–Poiseuille hydraulically weighted D_H_ (m).

The vulnerability index indicates a rough valuation about the trees sensitivity to the risks of cavitation. An increase in the V_X_ shows higher potential susceptibility of the hydraulic system to damages (Tyree and Zimmermann, 2002). The V_X_ was calculated as follows (Carlquist, 1977; Bauerle et al., 2011; Aref et al., 2013)

$$\begin{array}{l} V_{X}=\frac{D_{H}}{VD} \end{array}$$

where VD is vessel density and D_H_ is the Hagen–Poiseuille hydraulically weighted D_H_.

### Xylem Hydraulic Traits Time Series

For investigating the inter-annual variability of xylem hydraulic traits, we developed chronologies of D_H_, K_S_, and V_X_ for the three studied species following standard dendrochronological procedures. We observed an increasing trend in hydraulic traits' time series (Figure S2). It has been shown that D_H_ systematically increases with plant size, including plant height and correlated stem diameter in tropical woody species (Olson and Rosell, 2013) as well as on a global scale (Olson et al., 2014). To avoid any bias of increasing vessel size with increasing stem diameter, we removed age or size related trends from each hydraulic trait time series before chronology development. We used a cubic smoothing spline function with a 50% frequency response at 10 years for detrending, because we aimed at removing multi-decadal variability from our rather short (< 100 years) time series, and to retain high-frequency signals related to inter-annual climate variability. Detrended time series were then obtained by dividing the observed values by the fitted values (Briffa and Jones, 1990). The detrended time series of nine trees of each of the three species were averaged by a bi-weight robust mean to build the chronologies. The chronologies were build using the “chron” function in the Dendrochronology Program Library in R (dplR) (Bunn, 2008, 2010) within the R statistical programming environment (R Development Core Team, 2016). The robustness of the chronologies were assessed using standard statistics commonly used in dendrochronology including the mean inter-series correlation between trees (rbar.bt) (Briffa and Jones, 1990), the expressed population signal (EPS) (Wigley et al., 1984; Buras, 2017) and mean sensitivity (MS) (Briffa and Jones, 1990).

Intra-annual variability of wood anatomical parameters is also a research focus particularly in temperate regions. Such investigations require splitting of a ring into several sections. However, the number of the generally rather large vessels is very low in our studied species particularly in *T. ciliata* and *L. speciosa* (Figure 3). Splitting a ring into different sections might further reduce the vessel number per section due to splitting across some vessels. Particularly in narrow rings, sectioning is not feasible due to the very low number or even complete absence of vessels in some sections which does not provide adequate replication for statistical analysis. Hence, we refrained from intra-annual analysis of vessel features and instead considered the complete annual rings.

### Dendroclimatic and Statistical Analysis

Bootstrap correlation analysis was performed to assess the influence of climate variables (temperatures, T_mean_, T_min_, and T_max_; precipitation; Palmer Drought Severity index, PDSI; and Vapor Pressure Deficit, VPD) on xylem hydraulic traits. The bootstrap procedure generated 1,000 bootstrapped samples from the original hydraulic trait indices to test the significance of correlation coefficients and the stability of the error estimates more precisely (Guiot, 1991). We used the “bootRes” package in R to compute Pearson correlation coefficients between the hydraulic traits and each of the climatic parameters. A 20 months window from May of the previous year to current year December was used to correlate hydraulic conductivity time series with monthly climate data. Previous year's climate was included because current year's tree-ring features might be influenced by previous year climate through carbon carryover effects which is evident from the auto-correlation in the developed chronologies (0.09–0.56) (Fritts, 1976). One way analysis of variance (ANOVA) was performed to test the differences in hydraulic traits (D_H_, K_S_, and V_X_) among the three species. Requirements for normal distribution and statistical methods were checked. In order to assess the relations of hydraulic traits (D_H_, K_S_, and V_X_) with tree radial growth (RW) and various vessel features (MVA), we used simple linear regression analysis. To investigate the relationships between the hydraulically weighted D_H_ and VD we also used regression equation fitting exponential curve.

Temperature, precipitation, and humidity data were obtained from the Bangladesh Meteorological Department (BMD). PDSI data were collected from the KNMI climate explorer (Royal Netherlands Meteorological Institute) (https://climexp.knmi.nl/start.cgi). We calculated vapor pressure deficit (VPD) from the station data following the equations: VPD = [(100 - RH)/100]^*^SVP, where SVP = 0.610exp^*^[(17.27^*^T)/(T+237.3)], T = temperature, RH = relative humidity (Murray, 1967). All analyses were performed using different statistical packages within the R statistical programming environment (R Development Core Team, 2016)

## Results

### Interspecies Variation in Xylem Hydraulic Traits

**Figure 4:**
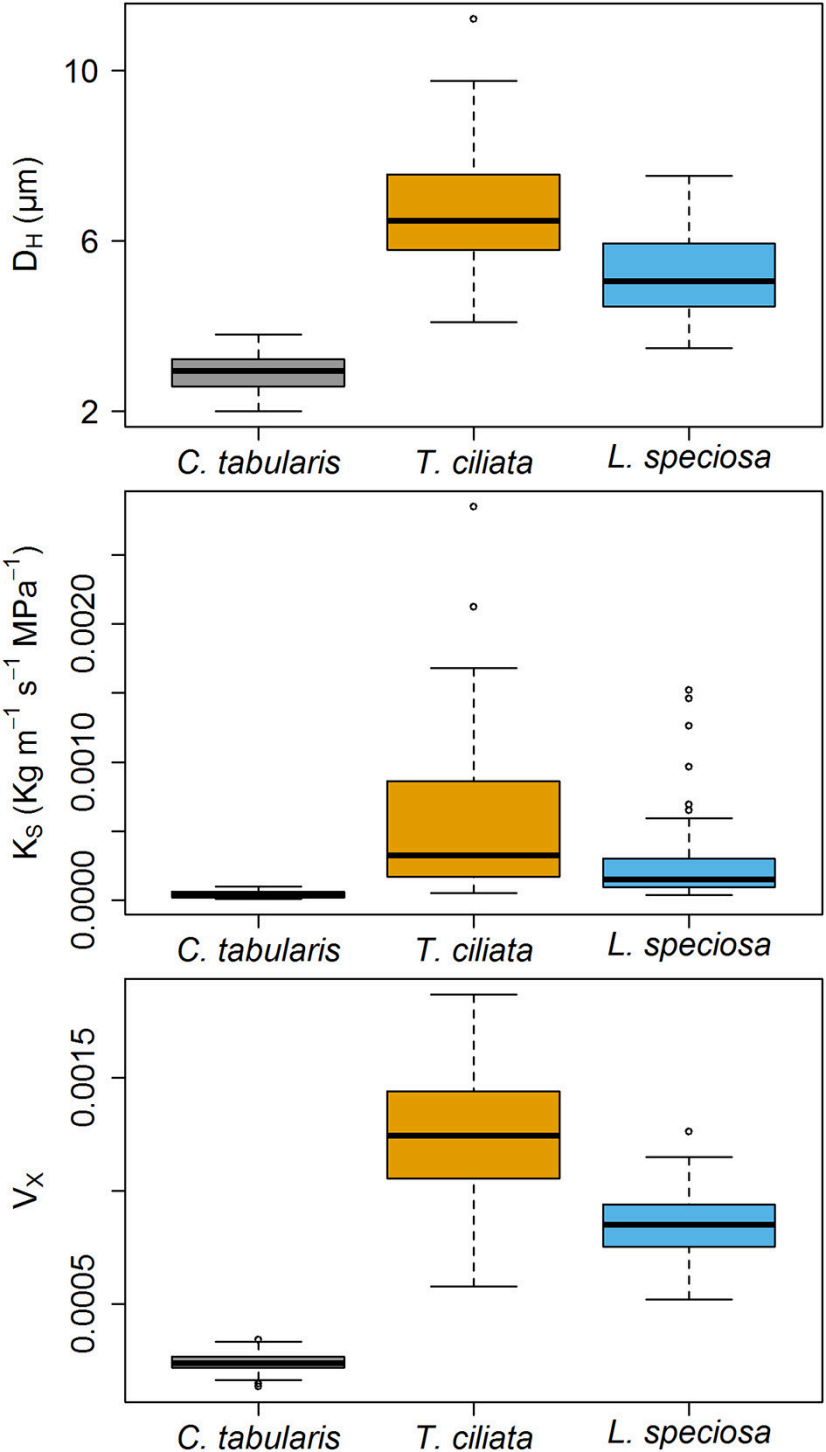
Boxplots of Hagen–Poiseuille hydraulically weighted vessel diameter (D_H_), Potential specific hydraulic conductivity (K_S_) and vulnerability index (V_X_) of three South Asian tropical moist forest tree species. Different letters indicate significant difference (*p* < 0.01) among the species.

Xylem hydraulic traits showed significant differences between species (Figure 4). Highest variations was observed in the hydraulic vulnarability to cavitaiton (V_X_) [*F*_(2, 243)_ = 582.51, *p* < 0.001], followed by the hydraulically weighted vessel diamter (D_H_) [*F*_(2, 243)_ = 294.67, *p* < 0.001]. The lowest but still strongly significant variation was observed in potential hydraulic conductivity (K_S_) [*F*_(2, 243)_ = 45.008, *p* < 0.001]. Intermediate shade tolerant and diffuse porous *C. tabularis* exhibited the lowest D_H_, K_S_, and V_X_ in comparison to the ring posours *T. ciliata* and *L. speciosa*. However, semi ring-porous and shade intolerant *T. ciliata* had higher D_H_, K_S_, and V_X_ than shade tolerant and ring-porous *L. speciosa* (Figure 4). Differences in D_H_, K_S_, and V_X_ showed a consistent pattern among the species.

### Xylem Hydraulic Traits Chronologies

**Figure 5:**
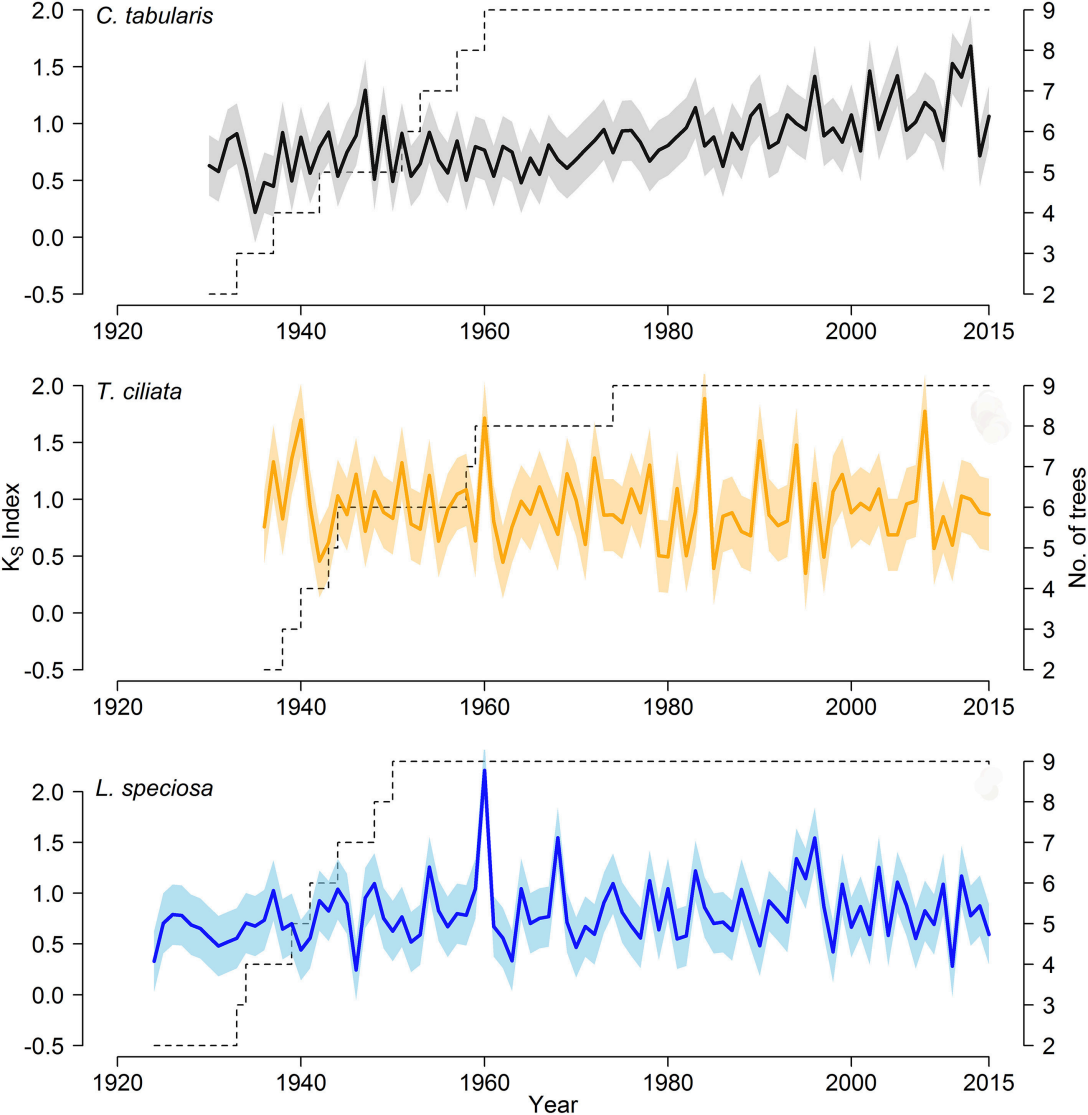
Standard chronologies of hydraulic conductivity (K_S_) of three South Asian tropical moist forest tree species; Color shading shows a ±1 SD interval around the means of the chronologies.

We developed three hydroulic trait chronologies for all the three species (Figure 5, Figure S1). The statistical parameters indicating the signal strength of the chronologies are described in Table 2. The raw tree ring series used to develop standard chronologies are also shown in Figure S2. Among the three chronologies within a species, specific hydraulic conductivity (K_S_) showed the highest common signal in all species as indicated by mean inter series correlation (rbar.bt) that varied between 0.17 and 0.22 among the species. The subsequent EPS was also highest in K_S_ among the three hydraulic traits with highest EPS in *Toona ciliata* (0.72). The lowest rbar.bt and EPS were observed in V_X_ chronology in all species. However, inter annual variation indicated by MS was high in all chronologies except D_H_. In all species, highest MS was found in K_S_. Based on the chronlogy statistics we progressed with K_S_ for further dendroclimatic analysis because K_S_ chronologies in all species showed the highest chronology signal which were consistent with values reported for wood anatomical time series (García-González et al., 2016). The K_s_ chronologies also provide indirect evidence of vulnerability to cavitation since hydraulic efficiency is directly related to hydraulic vulnerability (Markesteijn et al., 2011b).

**Table 2:** Chronology characteristics of three hydraulic traits for three South Asian moist tropical forest tree species.

**Species**	**Hydraulic trait**	AC1^*^	Mean GLK^*^	rbar.bt^*^	**Expressed population signal (EPS)**	**Mean sensitivity (MS)**
*C. tabularis*	D_H_	0.56	0.54	0.13	0.57	0.19
	K_S_	0.55	0.53	0.19	0.66	0.60
	V_X_	0.47	0.53	0.12	0.55	0.30
*T. ciliata*	D_H_	0.32	0.52	0.175	0.66	0.27
	K_S_	0.33	0.53	0.22	0.72	0.86
	V_X_	0.38	0.51	0.08	0.44	0.39
*L. Speciosa*	D_H_	0.32	0.51	0.172	0.65	0.24
	K_S_	0.09	0.52	0.172	0.65	0.77
	V_X_	0.27	0.49	0.02	0.16	0.35

*Chronology lengths of C. tabularis, T. cliliata and L. speciosa cover 85 years (1930–2015), 81 years (1934–2015), and 91 years (1924–2015), respectively. Mean series lengths (year) of C. tabularis, T. cliliata, and L. speciosa chronologies are 73, 69, and 80 years, respectively. For the description of abbreveations of hydraulic trait variables see Figure 4*.

* *AC1, 1st order autocorrelation; GLK, Gleichläufigkeit (sign test); rbar.bt, Mean inter-series correlation between trees*.

### Xylem Hydraulic Response to Climate Variability

**Figure 6:**
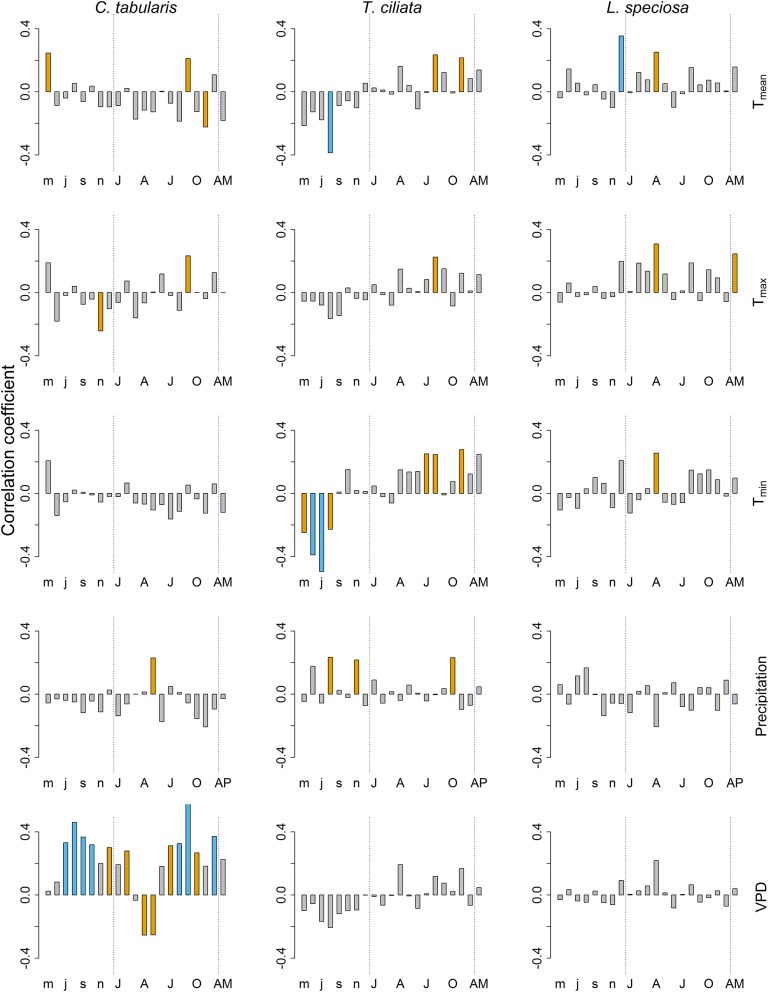
Bootstrap correlations between climate variables and hydraulic efficiency (K_S_) of three South Asian tropical moist forest tree species. Orange and blue bars indicate correlations significant at *p* < 0.05 and *p* < 0.01 levels, respectively. Lower case letters represent months of the previous year. Upper case letters represent months of the current year. AM, Annual mean; AP, Total annual precipitation.

Among the climate variables, VPD had the dominant influence on the variability of hydraulic conductivity (K_S_) in *C. tabularis* (Figure 6). Both current year and previous year monsoon and post monsoon season VPD had a positive impact on K_S_ whereas late spring and summer VPD negatively affected K_S_ in *C. tabularis* (highest correlation with current year September, *r* = 0.61, *p* < 0.001). K_S_ was significantly positively correlated with late monsoon season (September) mean and maximum temperatures (T_mean_, T_max_) and early monsoon season (May) precipitation (Figure 6). Previous year May temperatures also positively influenced K_S_ whereas temperature in the later growing season (November) negatively affected K_S_ in *C. tabularis*.

In *T.ciliata*, K_S_ was positively related to both day and night time temperatures (T_mean_, T_min_, and T_max_) and precipitation during the late monsoon season (Figure 6). In contrast, previous year temperatures during the main monsoon and late monsoon season were negatively related to K_S_, with a strong negative correlation with previous year August T_mean_ (*r* = −0.39, *p* < 0.01) and previous year June and July T_min_ (*r* = −0.49, *p* < 0.001). Previous year late monsoon precipitation positively affected K_S_.

K_S_ in *L. speciosa* was strongly and positively affected by previous year winter temperature mean temperature (December; *r* = 0.35, *p* < 0.01; Figure 6). Similarly, late spring and early summer temperatures (April) (T_mean_, T_min_, and T_max_) significantly positively influenced K_S._ Mean annual maximum temperature (T_max_) was also positively related to K_S_. Nonetheless, Precipitation had no significant influence on the K_S_ in *L. speciosa*. We found no significant correlation of K_S_ with PDSI in any of the studied species.

### Interrelationship Between Hydraulic Traits and Radial Growth

**Figure 7:**
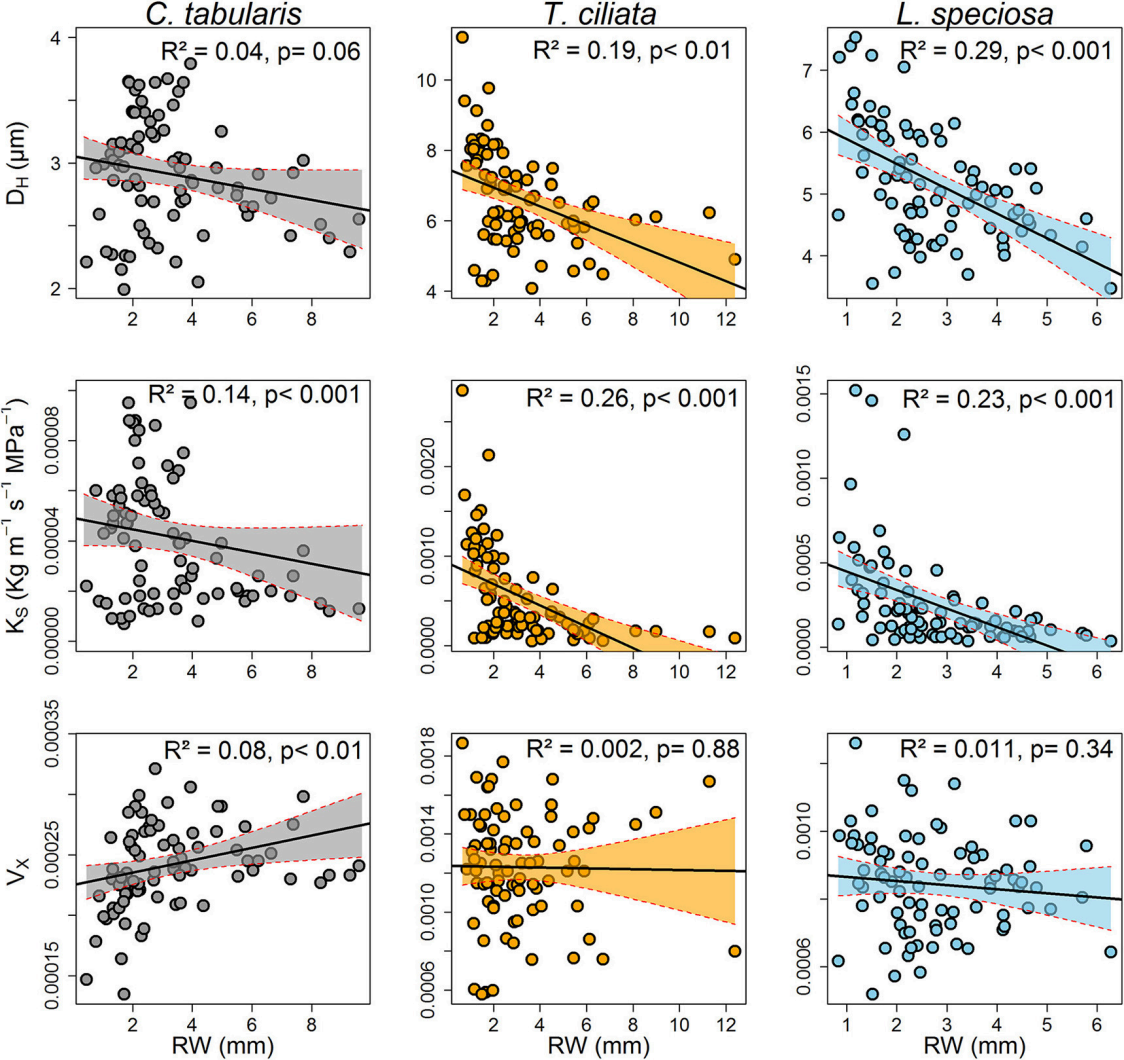
Relationships between ring-width and wood hydraulic traits of three South Asian tropical moist forest tree species. Each data point represents the mean of the respective years of 9 trees. Shaded areas represent 95% confidence intervals.

A strong linear and inverse relationship was found between radial growth and hydraulically weighted diameter (D_H_) in *T. ciliata* (*R*^2^ = 0.19, *p* < 0.01) and *L. speciosa* (*R*^2^ = 0.29, *p* < 0.01). However, the relationship was not significant in *C. tabularis* (Figure 7). The potential hydraulic conductivity (K_S_) was linearly positively related to radial growth in all three species, with the highest variance explained in *T. ciliata* (26%) followed by *L. speciosa* (23%).

**Figure 8:**
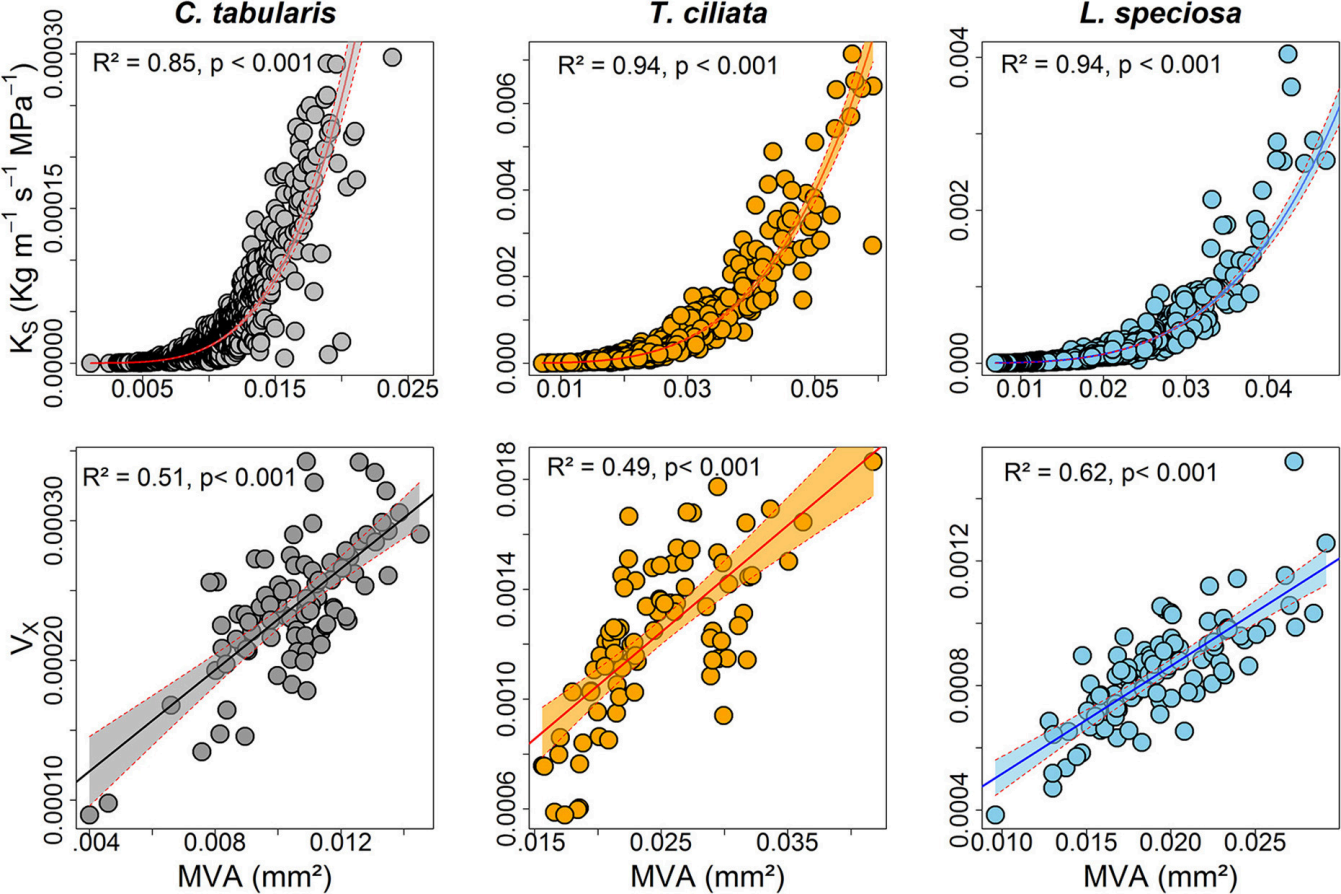
Relationships between hydraulic traits and vessel size (mm^2^) of three South Asian tropical forest tree species. Shaded areas represent 95% confidence intervals.

K_S_ was strongly positively connected to MVA in all the three species, explaining 85–94% of the variations. Likewise, hydraulic vulnerability strongly increased with MVA explaining the highest variation by *L. speciosa* (62%) (Figure 8). MVA was negatively associated with radial growth in two of the three species except *C. tabularis* (Figure S3). A strong negative relationship was found between radial growth and VD in all the studied species, with highest variation (56%) explained in *L. speciosa* (Figure S3). As we expected, hydraulic conductivity was positively related to hydraulic vulnerability in all species (*p* < 0.001) (Figure S4).

### Xylem Adjustment: Safety vs. Efficiency

**Figure 9:**
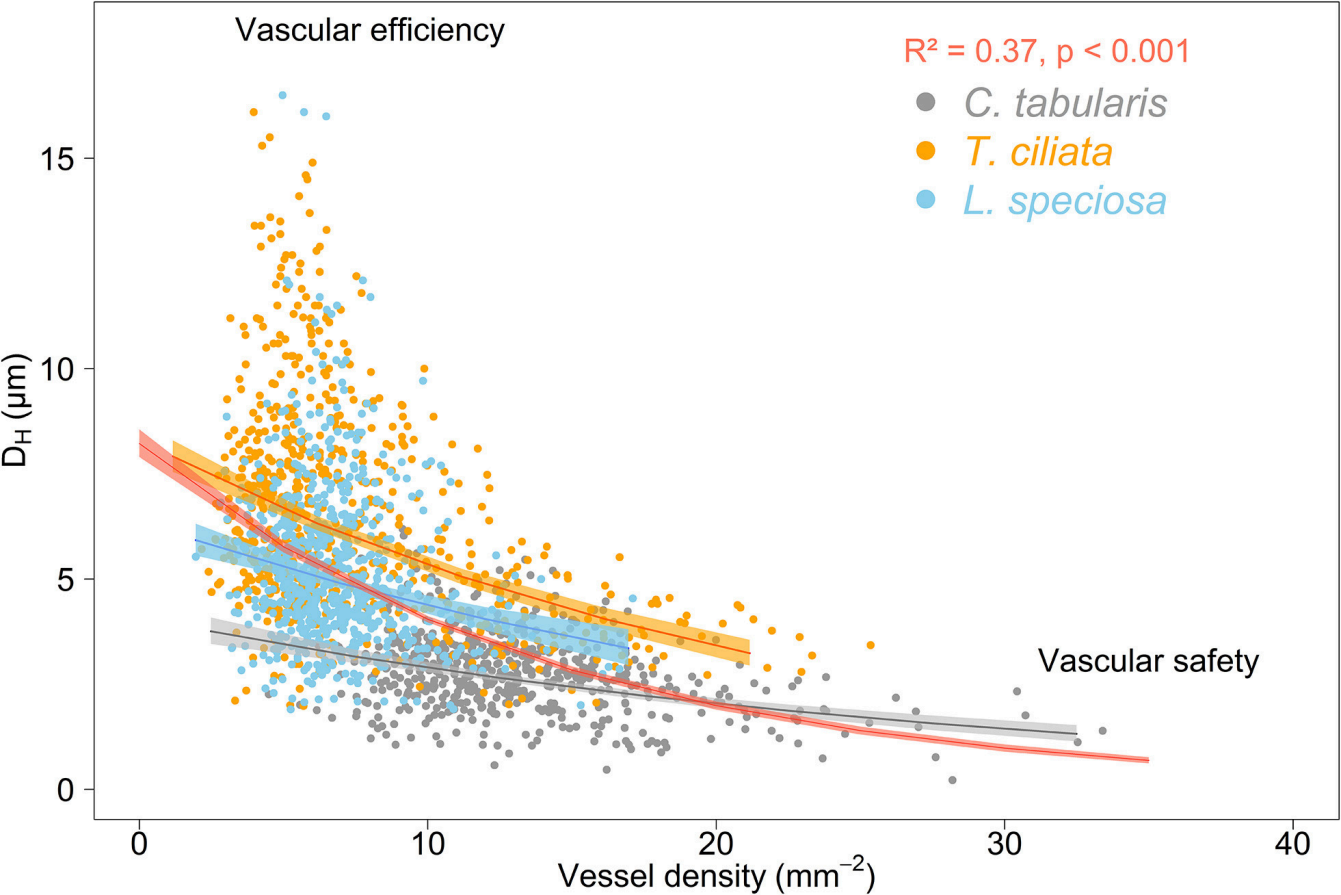
Trade-off between hydraulic efficiency and hydraulic safety in three South Asian tropical moist forest tree species. The red curve represents the average of all 27 measured trees. Shaded areas represent 95% confidence intervals.

We observed the commonly expected trade-off between hydraulically weighted D_H_ and VD. VD increased when D_H_ decreased, leading to decreased vulnerability to hydraulic failure due to cavitation. This inverse relation was stronger when analyzed on the tree community level (*R*^2^ = 0.37, *p* < 0.001) than on the species level (trees within a species) (Figure 9). Ring-porous *T. ciliata* and *L. speciosa* exhibited a stronger trade-off between D_H_ and VD than diffuse porous *C. tabularis*. The variation explained by the inverse relationship of D_H_ and VD ranged between 5 and 21% among the three species, with *T. ciliata* explaining the highest variation.

## Discussion

### Long Term Hydraulic Adjustment to Climate Variability

We compared standard chronologies of specific hydraulic conductivity (K_S_) with climate variables to evaluate the long-term hydraulic adjustment to climate variability. The robustness of the K_S_ chronologies for dendroclimatic analysis was reflected in the chronology statistics because Ks displayed the highest values in all the statistical parameters among the three chronologies in all three species. Although the common signal was rather low and did not pass the recommended threshold for the EPS statistic of 0.85 (Table 2), the values were within the normal range commonly found in wood anatomical time series (García-González et al., 2016). Our chronology statistics are consistent with hydraulic conductivity (K_S_) time series of other ring-porous (Corcuera et al., 2006; Campelo et al., 2010; Gea-Izquierdo et al., 2012; Pérez-de-Lis et al., 2018) and diffuse-porous (Corcuera et al., 2004b; Oladi et al., 2014; Rita et al., 2015; Schuldt et al., 2016; Noyer et al., 2017) broadleaf trees. Despite of the low common signal, the chronologies exhibited strong relationships with climate variables. Our findings are in line with other studies which reported low common signals but a high correlation with climate in a wide range of species and forest types (Pumijumnong and Park, 1999; García-González and Eckstein, 2003; Fonti et al., 2007; Campelo et al., 2010; González-González et al., 2013; Pritzkow et al., 2016; Martínez-Sancho et al., 2017). Despite of species specific climate responses, we observed some common climate influence on all three species in spring and summer (pre-monsoon) season.

Overall, hydraulic conductivity (K_S_) of all species positively responded to current year temperatures, precipitation and VPD except the inverse relation of K_S_ in *C. tabularis* and April-May VPD. VPD in association with mean and minimum temperatures exerted dominant control over the xylem water transportation capacity in *C. tabularis*, whereas *T. ciliata* was affected by both temperature and precipitation. Temperature had the dominant effect on the hydraulic conductivity (K_S_) of *L. speciosa*. Higher temperature increases VPD, leading to increased evapotranspiration. In order to response to high evaporative demand, trees maximize their water transport efficiency. The underlying mechanism of this increased hydraulic conductivity (K_S_) lies in the fact that high temperature impairs the cell differentiation processes, particularly during latewood formation. Occasionally, during the post-monsoon season, latewood formation is completely ceased in response to high evaporative demand, which results in a ring with a small number of large size earlywood vessels. Consequently, the MVA increased as an adjustment to support efficient water transport. However, previous year temperatures had a negative influence on hydraulic conductivity (K_S_). Higher temperatures limited tree growth in the previous year, facilitating storage of carbohydrate reserves which create favorable conditions for tree growth of the following year (Fritts, 1976). This results in a reduced conductivity in the following year since small size latewood vessels reduce mean hydraulically weighted diameter and MVA. The positive influence of precipitation on conductivity is most likely caused by growth reduction due to high soil moisture content, particularly in the main monsoon months and in the post monsoon season (Rahman et al., 2018). Both *C. tabularis* and *T. ciliata* are sensitive to water saturation since they prefer well-drained soil conditions (Orwa et al., 2009).

Vulnerability to cavitation is an important hydraulic trait which determines the competitiveness of a particular species under climatic stress. Due to a lower common signal in V_X_ chronologies we were unable to compare them with climate variables. However, we observed that higher hydraulic conductivity (K_S_) increased hydraulic vulnerability in all species (Figure S4). Consistent with our results many studies have found the positive relationship between hydraulic conductivity and vulnerability (Markesteijn et al., 2011a; Oladi et al., 2014; Pérez-de-Lis et al., 2018). It is also noted that trunk level hydraulic conductivity may be confounded by whole tree architecture such as leaf area to sapwood area and may vary along a height gradient from root to shoot (Kotowska et al., 2015). Considering these factors however, requires huge time and budget and we hope to take them in to account in our next project.

### Inter Specific Variations in Phenotypic Plasticity

Plants may show differential growth and wood anatomical adjustment to stressful environmental conditions. The adjustment mechanism may vary depending on tree functional types and their life history strategies (Poorter et al., 2010; Markesteijn et al., 2011a). In our study, semi ring-porous and ring-porous *T. ciliata* and *L. speciosa* showed similar responses to water stress, maintaining an inverse relation of radial growth with hydraulically weighted diameter (D_H_) and MVA (Figure 7 and Figure S3). By the way of increasing D_H_, ring-porous trees increase the efficiency of water transportation as reflected in increased potential specific hydraulic conductivity (K_S_). When radial growth decreases during the late growing season, more carbohydrate reserves may be available for earlywood formation in the following year (Lacointe, 2000; Richburg, 2005). Simultaneously, vessel frequency increased to keep a higher number of smaller vessels hydraulically functional and thus to avoid drought induced cavitation (Sperry et al., 2008; Zanne et al., 2010; Venturas et al., 2017; Pérez-de-Lis et al., 2018). Our results are in line with studies on ring-porous tree species in both temperate (Gea-Izquierdo et al., 2012; Pérez-de-Lis et al., 2018) and Mediterranean forests (Corcuera et al., 2006; Gea-Izquierdo et al., 2012; Rita et al., 2016; Castagneri et al., 2017; Martínez-Sancho et al., 2017). Some studies relating hydraulic traits with life history strategies in tropical regions also yielded similar adjustment mechanisms as reported here (Poorter et al., 2010; Zanne et al., 2010; Markesteijn et al., 2011a; Fan et al., 2012).

In contrast, diffuse porous *C. tabularis* did not show an increase in water transport efficiency since D_H_ and MVA did not significantly increase in water stress conditions, although vessel frequency increased to reduce the risk of hydraulic failure (Figure 7 and Figure S3). The facts that *C. tabularis* maintains a constant D_H_ and MVA under water stress and shows a strong linear positive relation of MVA to V_X_ (Figure 8), suggest that the species is less vulnerable to cavitation risk than the ring-porous species we studied. Hence, the species specific differences in D_H_, K_S_, and V_X_ (Figure 4) clearly support differences in adjustment strategies between ring-porous and diffuse porous trees in our study. In line with our results, diffuse porous *Fagus sylvatica* and *Fagus orientalis* showed similar plasticity in order to avoid hydraulic failure due to water stress in temperate forests (Pourtahmasi et al., 2011; Rita et al., 2015; Schuldt et al., 2016; Noyer et al., 2017). Ring-porous and shade tolerant *L. speciosa* was found to be more effective to prevent hydraulic failure under drought conditions than the shade intolerant *T. ciliata*. The above findings suggest that ring-porous species are more efficient in water transport than the diffuse porous trees, whereas diffuse porous trees are less vulnerable to cavitation under water stress, as it is clearly reflected in mean K_S_ and V_X_ indices of our studied species (Figure 4).

### Does Higher Hydraulic Efficiency Favor Tree Radial Growth?

Efficient water transport is usually expected to favor tree radial growth (Zanne et al., 2010). Numerous studies confirmed the positive association between hydraulic conductivity and tree radial growth across major biomes in the world (Fichot et al., 2009; Zanne et al., 2009; Poorter et al., 2010; Markesteijn et al., 2011a; Fan et al., 2012; Gleason et al., 2012; Hoeber et al., 2014; Rungwattana and Hietz, 2017). Very recently, Hietz et al. (2017) analyzed a larger data set of 325 species from a Panamanian rainforest and revealed that hydraulic conductivity is the best predictor of biomass growth rates. The probable mechanism underlying this positive relationship is attributed to higher leaf level photosynthetic rate due to efficient water transport (Meinzer et al., 2008; Choat et al., 2012). Higher hydraulic efficiency results in plant tissues comprised of higher ratio of vessel lumen area which has lower construction and maintenance costs, which may also explain the positive alignment of hydraulic conductivity to radial growth (Gleason et al., 2016).

Interestingly, we observed a negative relationship between hydraulic conductivity and tree radial growth in all species, with *L. speciosa* showing the strongest negative relation (Figure 7). However, our results are consistent with findings from many other ring-porous (Gea-Izquierdo et al., 2012; Rita et al., 2016) and diffuse porous species (Oladi et al., 2014; Rita et al., 2015). Our studied species responded to the drought stress conditions (e.g., higher temperature, higher evaporative demand, and higher VPD) by slowing down the cell differentiation process (reduced radial growth), and maintaining plasticity in hydraulic architecture such as increased vessel frequency as a safety mechanism against cavitation. Under stress, carbohydrate accumulation is highly favored rather than investing in biomass growth, which leads to reduced radial growth but following year tree radial growth might be stimulated by the accumulated carbohydrate reserve (Lacointe, 2000; Richburg, 2005). Nonetheless, we cautiously interpret the differences in the conductivity-growth relationships observed in our study from that in other studies which mainly focused on conductivity over the whole lifespan rather than to year-to-year variations. Our findings and the evidence of other studies, however, suggest that the widely expected conductivity-productivity positive relationship may not be consistent and calls for further studies.

### Xylem Safety vs. Efficiency: How Consistent at Tree and Species Level Under Humid Environment?

Trees respond to stress by drought or higher VPD by producing a higher number of small vessels to reduce the risk of cavitation (hydraulic safety) and reduce maximum vessel diameters to maintain the efficiency of water transpiration (hydraulic efficiency) (Sperry et al., 2008; Hacke et al., 2017; Haworth et al., 2017; Venturas et al., 2017; Pérez-de-Lis et al., 2018). However, proper knowledge whether this safety-efficiency trade-off occurs only within one individual in response to short-term variations to drought, or whether this trade-off is consistent between trees of the same species or even between individuals of different species is particularly important in the context of long-term global environmental change adaptability. We analyzed hydraulically weighted D_H_ and vessel frequency data derived from a total of 1,429 tree rings of three species over the past nine decades. Our analysis confirmed the well-known trade-off between hydraulic conductivity and safety both in trees within and between species for a long-term (Figure 9). The species-specific hydraulic efficiency-safety relationships fitted exponential functions which reached lower *R*^2^ values than a function fitted through the community of studied trees of all species.

Consistent with our results, many studies confirmed this trade-off across temperate (Zanne et al., 2010), Mediterranean (LoGullo and Salleo, 1988; Tognetti et al., 1999; Martínez-Sancho et al., 2017), riparian (Pockman and Sperry, 2000), and tropical tree species (Poorter et al., 2010; Fan et al., 2017; Hietz et al., 2017), although long-term analyses are still absent for the tropics. Many studies found a week or even no trade-off between hydraulic conductivity and hydraulic safety (Maherali et al., 2004; Westoby and Wright, 2006; Gleason et al., 2016; Schuldt et al., 2016) which implies that this relationship is variable. By analyzing a large data set, Gleason et al. (2016) found that many woody species showed low safety and low efficiency, but no acceptable causes were attributed after they have analyzed this trade-off in terms of both species level traits and climate variables at different spatial scales. Gleason et al. (2016) (and references therein) also stated that micro anatomical features such as pit membrane structure or conduit length could be relevant to determine xylem safety although there features were not captured in vessel anatomy parameters measured in cross-sections. Nevertheless, species and climate specific long-term adjustment mechanisms as we investigated in this study can provide important insight into how a particular tree species and its populations will respond to environmental stress in a particular ecosystem.

## Conclusions

Overall, we observed differential responses and adjustment mechanisms of hydraulic behavior to climatic stress varying with tree life history strategies and wood traits. The hydraulic adjustment however could not avoid reduced tree growth in any of the three species we studied despite of their functional differences. Growing conditions in our study region are expected to getting worse due to ever increasing temperature and higher frequency of drought and other extreme events. It is however not clear to what extent tropical trees will be able to adjust their hydraulic system under future global changes. To get more insight in to the possible future hydraulic response of tropical trees, such studies should be extended to additional species and sites across the tropics. Future research should also consider intra-annual variations of hydraulic traits which might allow improving our understanding of the seasonality and associated mechanisms driving the hydraulic behavior of tropical trees.

## Author Contributions

MI and AB designed the study. MI and MR conducted field work and prepared samples for wood anatomical measurements. MI performed wood anatomical measurements and analyzed data. MI, MR, and AB interpreted the results and wrote the manuscript.

### Conflict of Interest Statement

The authors declare that the research was conducted in the absence of any commercial or financial relationships that could be construed as a potential conflict of interest. The handling Editor declared a past co-authorship with one of the authors AB.
