# Supplementary material for: Long-Term Hydraulic Adjustment of Three Tropical Moist Forest Tree Species to Changing Climate
Source: Front Plant Sci. 2018 Dec 4;9:1761. doi: 10.3389/fpls.2018.01761 (PMC6288455; doi:10.3389/fpls.2018.01761)
Supplement: Supplementary file 1 [file Table_1.DOCX]

**Supplementary materials**

**Long-term hydraulic adjustment of three tropical moist forest tree species to changing climate**

Mahmuda Islam^1,2*^, Mizanur Rahman^1,2^, Achim Bräuning^1^


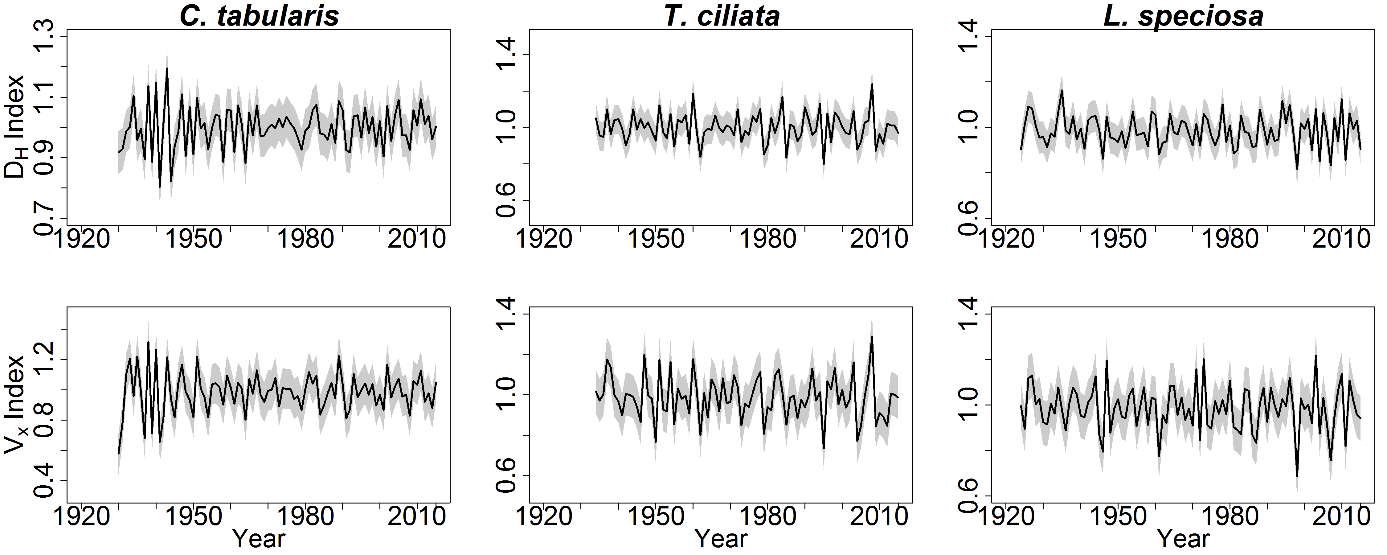


Figure S1. Standard chronologies of Hagen–Poiseuille hydraulically weighted diamter (D_H_), hydraulic resistivity (r_s_), and hydraulic vulnerability (V_X_) of three South Asian tropical moist forest tree species; Shading areas show ±1 SD around the means of the chronologies.


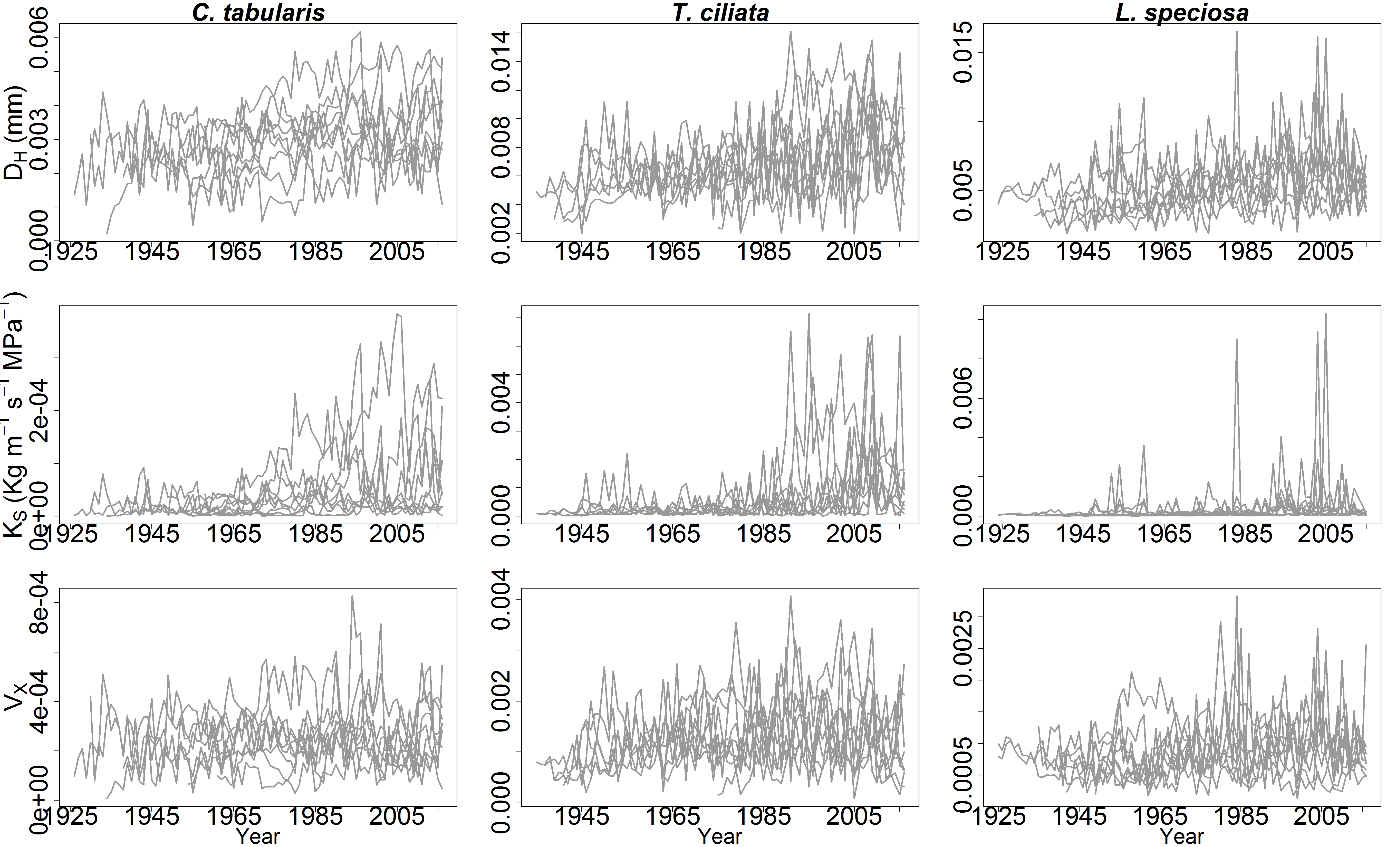


Figure S2. Raw series of Hagen–Poiseuille hydraulically weighted vessel diameter (D_H_), potential specific hydraulic conductivity (K_S_) and vulnerability index (V_X_) of three South Asian tropical moist forest tree species.

***
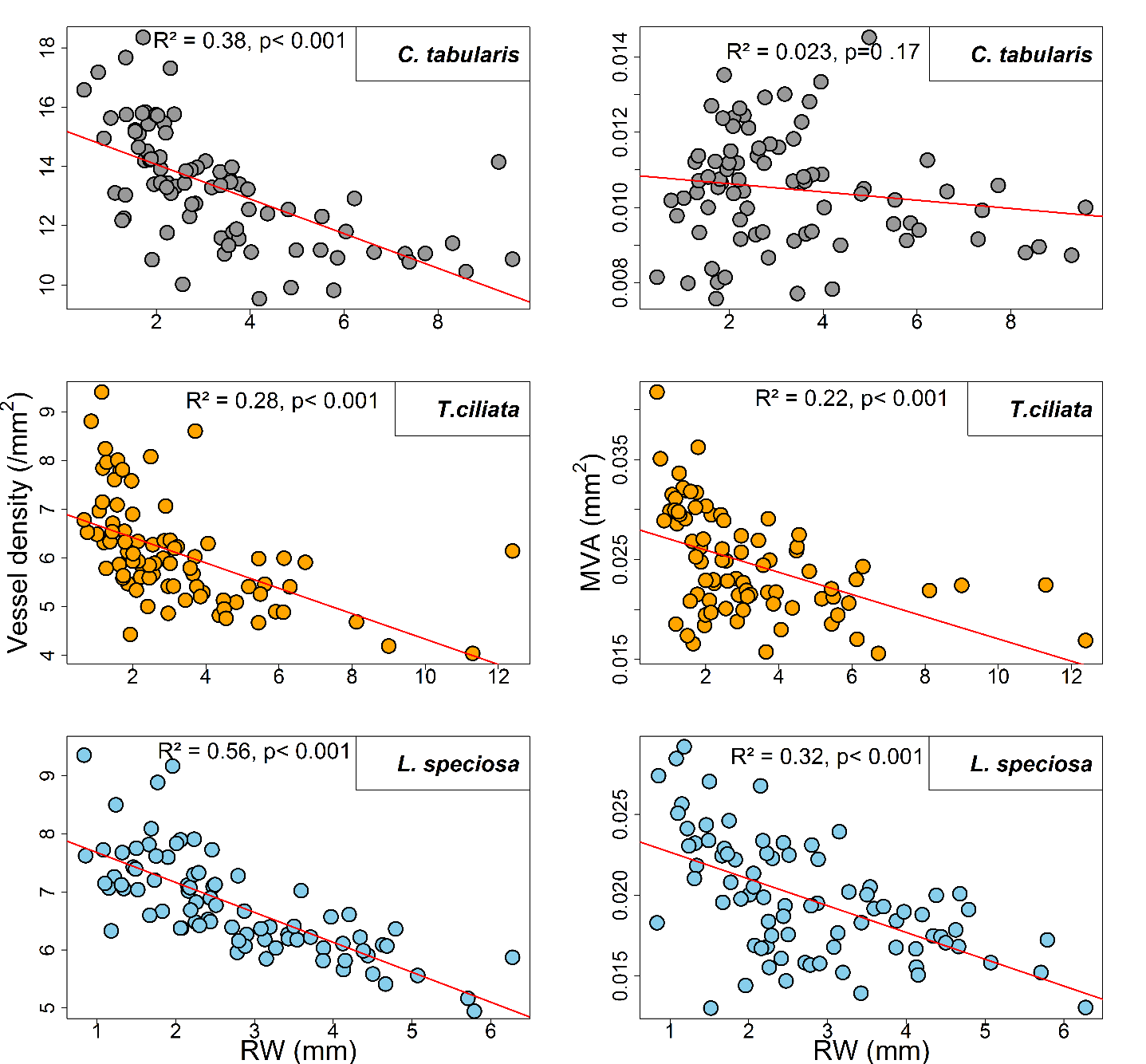
***

Figure S3. Relationships between ring-width and vessel parameters of three South Asian tropical moist forest tree species.


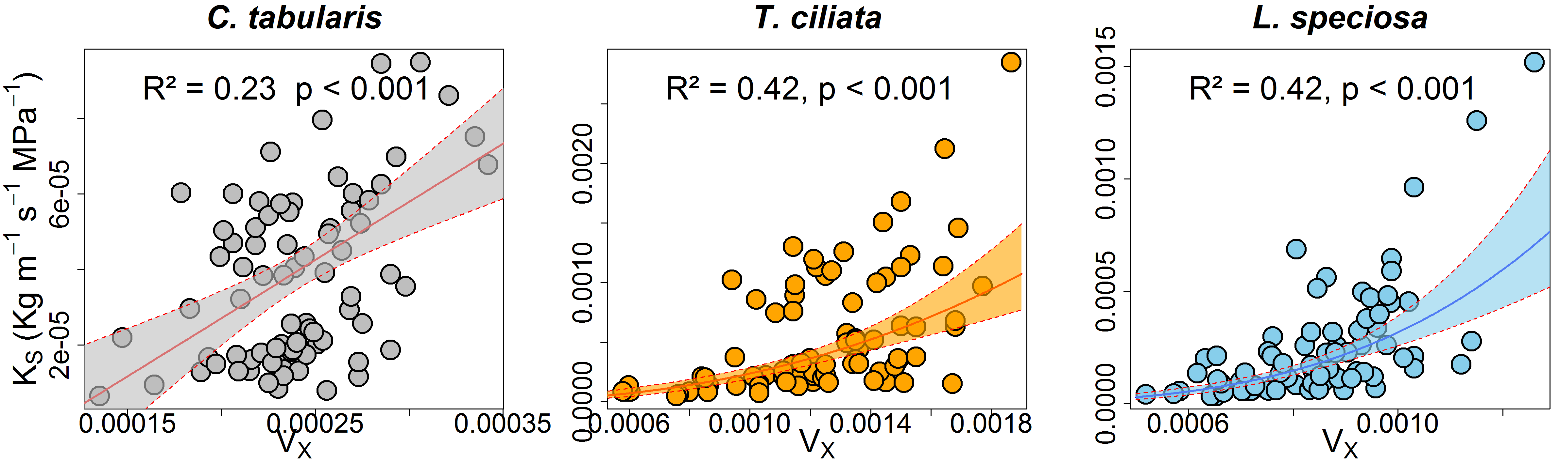


Figure S4. Relationships between potential specific hydraulic conductivity (K_S_) and vulnerability index (V_X_) of three South Asian tropical moist forest tree species.
